# Pharmacologic Evaluation of Antidepressant Activity and Synthesis of 2-Morpholino-5-phenyl-6*H*-1,3,4-thiadiazine Hydrobromide

**DOI:** 10.3390/ph9020027

**Published:** 2016-05-19

**Authors:** Alexey P. Sarapultsev, Oleg N. Chupakhin, Petr A. Sarapultsev, Larisa P. Sidorova, Tatiana A. Tseitler

**Affiliations:** 1 Ural Federal University named after the first President of Russia B. N. Yeltsin, Ekaterinburg 620002, Russia; chupakhin@ios.uran.ru (O.N.C.); p.sarapultsev@gmail.com (P.A.S.); vlapp@isnet.ru (L.P.S.); tseitler85@mail.ru (T.A.T.); 2 Institute of Immunology and Physiology of the Ural Branch of the RAS, Ekaterinburg 620219, Russia; 3 The IJ Postovsky Institute of Organic Synthesis of the Ural Branch of the RAS, Ekaterinburg 620990, Russia

**Keywords:** adrenoblockers, α-adrenoblockers, heterocycles, thiadiazines, 1,3,4-thiadiazine-2-amines, serotonin, stress, pharmacological evaluation

## Abstract

Substituted thiadiazines exert a reliable therapeutic effect in treating stress, and a schematic description of their ability to influence all aspects of a stress response has been depicted. This study was conducted to pharmacologically evaluate compound **L-17**, a substituted thiadiazine, (2-morpholino-5-phenyl-6*H*-1,3,4-thiadiazine, hydrobromide) for possible anti-psychotic/antidepressant activity. Compound **L-17** was synthesized by cyclocondensation of α-bromoacetophenone with the original morpholine-4-carbothionic acid hydrazide. Pharmacologic evaluations were conducted using methods described by E.F. Lavretskaya (1985), and in accordance with published guidelines for studying drugs for neuroleptic activity. Compound **L-17** was evaluated for various possible mechanisms of action, including its effects on cholinergic system agonists/antagonists, dopaminergic neurotransmission, the adrenergic system, and 5-HT3 serotonin receptors. One or more of these mechanisms may be responsible for the beneficial effects shown by thiadiazine compounds in experiments conducted to evaluate their activity in models of acute stress and acute myocardial infarction.

## 1. Introduction

A variety of biological activities have been reported for a large number of thiadiazine derivatives, such as antimicrobial [1,2,3,4] antiagregant [5] anti-inflammatory and analgesic [6] and tuberculostatic activity [7,8]. Moreover, *in vivo* studies have shown their significant therapeutic effects in various models of disease, including models of systemic inflammation [9], myocardial infarction [10,11], and acute pancreatitis [12]. Besides, some compounds from the group of substituted thiadiazines were suggested to influence of all components of a stress-induced response [13]. Results of the previously mentioned experiments led us to hypothesize the existence of a central mechanism of action for substituted thiadiazines, and suggest this class of compounds as a promising source of new drugs to treat stress and diseases in which inflammation and stress play a major role, such as myocardial infarction and stress-induced cardiomyopathy.

**Figure 1:**
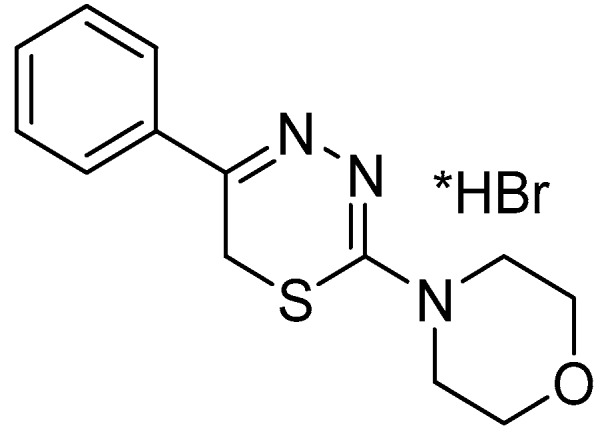
Compound **L-17**, a 5-phenyl substituted-6*H*-1,3,4-thiadiazine-2-amine.

This study was conducted to evaluate the substituted thiadiazine compound **L-17** (Figure 1) for possible anti-psychotic/anti-depressant activity.

## 2. Results

The results of our studies evaluating the overall effects of test drugs on exploratory/locomotor activity, fear response, and affective aggression in animals are shown in Table 1 and Table 2.

Our results (Table 1) showed that administration of compound **L-17** resulted in decreased animal motor activity (3-fold and > 20-fold decreases at doses of 60 mg/kg and 120 mg/kg, respectively), and an increase in the threshold voltage (to 43 ± 1.5 V and 48 ± 1.5 V when administered at doses of 60 mg/kg and 120 mg/kg, respectively). Squeak intensity (the equivalent of an "emotional" fear response) changed significantly following administration of compound **L-17** at a dose of 120 mg/kg.

Administration of Eglonyl led to a significant decrease in overall exploratory/locomotor activity (1.6-fold at a dose of 40 mg/kg), an increase in the threshold voltage (to 35 ± 0.5 V and 38 ± 0.6 V when administered at doses of 20 mg/kg and 40 mg/kg, respectively), as well as changes in squeak intensity (at a dose of 20 mg/kg).

Thus, administration of compound **L-17** led to the appearance of CNS-associated signs of depression similar to those induced by Eglonyl, which causes changes in spontaneous behavior (activity reduction), altered reactions to painful stimuli, and an increased threshold for a pain reaction. Compound **L-17** produced an analgesic effect when given at a dose of 10 mg/kg, while a 120 mg/kg dose induced a cataleptic state.

Changes in apomorphine-induced stereotypy were evaluated in mice (Table 2).

Animals that were administered **L-17** displayed a significant increase in their latent period starting at a 30 mg/kg dose, and also a reduced duration of stereotypy starting at a 60 mg/kg dose. The estimated effective dose (ED50) of **L-17** was 80 mg/kg. Animals which received the antipsychotic drug Eglonyl displayed significant increases in both their latent period and duration of stereotypy. The estimated effective dose (ED50) of Eglonyl was 19 mg/kg.

This test is specific to neuroleptic agents derived from phenothiazine and butyrophenone. An ability to suppress stereotypy indicates that the test substance inhibits dopaminergic neurotransmission in the nigrostriatal system of the brain. This trait was displayed by compound **L-17**.

Next, apomorphine-induced hypoactivity was evaluated in rats (Table 3).

Our results showed that compound **L-17** could potentiate the effects of presynaptic doses of apomorphine, and significantly reduce the motor activity of animals. A 40 mg/kg dose of **L-17** administered in conjunction with apomorphine (0.15 mg/kg) reduced animal motor activity by 26.4 ± 3.8 movements when compared with a control group (217.75 ± 17.10 movements) and also when compared with a group of animals administered apomorphine alone at a dose of 0.15 mg/kg (75.88 ± 19.33 movements). In contrast, a 40 m/kg dose of Eglonyl induced an increase in locomotor activity (152.38 ± 12.42 movements) when compared locomotor activity displayed by animals treated with apomorphine alone (75.88 ± 19.33 movements).

The amphetamine-induced locomotion test was conducted with mice (Table 4).

A 100 mg/kg dose of compound **L-17** produced a significant increase in the duration of an animal’s latent period, and a 200 mg/kg dose produced a significant decrease in stereotypy. The estimated effective dose (ED50) of compound **L-17** was 170 mg/kg. A 40 mg/kg dose of Eglonyl induced a significant decrease in the duration of stereotypy (40.3 ± 1.8 min) when compared with the duration in control animals (118.0 ± 1.2 min).

Liver enzyme activity was evaluated by using the hexenal test to examine the hypnotic effect of a barbiturate (hexenal) in mice (Table 5). Our results showed that a 60 mg/kg dose of compound **L-17** significantly increased the duration of both latency (up to 254.0 ± 75.0 s) and sleep (65.7 ± 8.1 min), when compared with those parameters in a control group (126.3 ± 6.4 sec and 39.3 ± 6.4 min, respectively). The estimated effective dose (ED50) of **L-17** was 80 mg/kg. A 25 mg/kg dose of Eglonyl produced an increase in sleep duration (up to 39.8 ± 1.7 min) when compared with that in control animals (25.0 ± 0.8 min), and the estimated ED50 of Eglonil was 42 mg/kg.

As antipsychotic drugs typically potentiate the narcotic effect of barbiturates [14], we also tested substances for their influence on the hypnogenetic effects of barbiturates (Table 5).

The results shown in Table 5 indicate that administration of a sub-threshold dose of hexenal caused 16.7% of the laboratory animals to fall asleep, while a 10 mg/kg dose of **L-17** administered in addition to hexenol caused a significantly greater percentage of the animals to fall asleep. This result suggests that compound **L-17** can decrease the ability of liver microsomes to metabolize barbiturates, which is a characteristic feature of many neuroleptics [14].

The arecoline-evoked tremor was evaluated in mice. The results shown in Table 6 indicate that a high dose (60 mg/kg) of compound **L-17** caused a significant reduction in the mean duration of tremor (14 ± 0.7 s), when compared with the mean duration in a control group; providing evidence for its anti-cholinergic effect [15,16].

The influence of compound **L-17** on the contractile activity of isolated smooth muscles (guinea pig ileum and rat seminal vesicles) was studied using acetylcholine and epinephrine in the standard method described by R. Blattner (Table 7) [17].

When injected at a concentration of 1 × 10^−5^ mol/L, compound **L-17** demonstrated a moderate ability to block adrenoreceptors, which indicated its ability to interact with adrenergic receptors, and in particular, alpha-1-adrenoceptors. Eglonyl blocked adrenoceptors when injected at a concentration of 1 × 10^−6^ mol/L. The ability of compound **L-17** to interact with 5-HT3 receptors was demonstrated in the model of serotonin-induced spasm of isolated guinea pig ileum muscle [18]. Significant reductions in contraction amplitude were observed after injecting either compound **L-17** or Eglonyl at a concentration of 1 × 10^−5^ mol/L.

Thus, our data suggest that compound **L-17**, which was selected from a group of 5-phenyl substituted-6*H*-1,3,4-thiadiazine-2-amines, might exert its effects via one or more mechanisms of action. These include affecting cholinergic system agonists/antagonists (arecoline 15 mg/kg), dopaminergic neurotransmission (apomorphine 0.1 mg/kg and amphetamine 5 mg/kg), the adrenergic system (evaluation of smooth muscle contractile activity), and (or) 5-HT3 serotonin receptors (model of serotonin-induced muscle spasm).

## 3. Discussion

The results shown in Table 1, Table 2, Table 3, Table 4, Table 5, Table 6 and Table 7 and by assays used to differentiate groups of drugs in initial screening tests [11] indicated that compound **L-17**, selected from a group of 5-phenyl substituted-6*H*-1,3,4-thiadiazine-2-amines, possesses a combination of adrenergic properties, as well as certain characteristics of choline and serotonin blockers. Compound 17 demonstrated its effects when used in a concentration range similar to that employed when using the atypical antipsychotic agent Eglonyl (sulpiride), as well as antidepressants such as amitriptyline, and alpha-blockers such as pyrroxanum.

Practically all effective anti-psychotic medications are known to block a1-adrenoreceptors [19]. While these receptors were initially thought to merely promote sedation, more recent data indicate that the ability to blockade a1-adrenoceptors contributes to the therapeutic effects anti-psychotic agents [19]. A1-adrenoceptors are believed to play a regulatory role in the brain by enhancing certain excitatory afferent inputs to brain cells. The first evidence for tonic regulation of serotonin neurons (5-HT) by postsynaptic alpha-1-adrenergic receptors belonging to the noradrenergic system was reported by TH Svensson in 1975 [20]. Those data were subsequently supported by several studies showing that alpha-1-blockers suppressed the activity of serotonergic neurons [21,22], and reduced serotonin levels in the hippocampus [23]. These findings explain why medications that act on serotonin and noradrenaline systems demonstrate their most pronounced activity as antidepressants [19]. Moreover, their synergistic effect may be due to the fact that alpha-1 and serotonin (5HT2A) receptors are involved in the phosphatidyl inositol/protein kinase C intracellular pathway via Gq proteins [24].

At the same time, it should be noted that the affinity of norepinephrine for alpha-1 adrenoceptors is significantly less than its affinity for alpha-2-adrenoceptors. As a result, a substantial increase in norepinephrine levels (e.g., as induced by a stress reaction) is required for norepinephrine to interact with alpha-1-receptors [19]. This explains why administration of an alpha-1-adrenergic receptor’s antagonist produces almost no effect under normal (non-stress) conditions, but has a protective effect during stressful conditions [25].

The mechanisms described above may be responsible for the beneficial effects of thiadiazine compounds, as previously demonstrated in experimental models of acute stress [13] and acute myocardial infarction [10,11].

## 4. Materials and Methods

### 4.1. Chemical Synthesis

**Figure 2:**
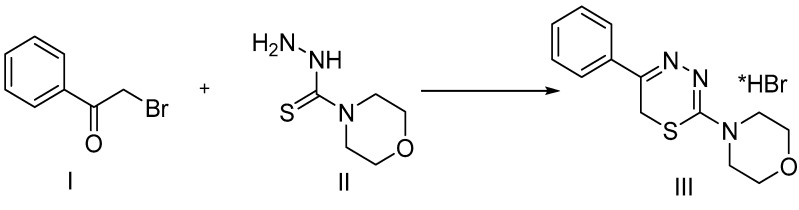
Chemical synthesis of thiadiazine derivatives.

Work on the synthesis of 1,3,4-thiadiazine derivatives has been ongoing for several decades at Ural Federal State University in Russia (Figure 2) [26,27,28,29].

The molecule 2-morpholino-5-phenyl-6*H*-1,3,4-thiadiazine hydrobromide (compound **L-17**) (**III**) was synthesized by cyclocondensation of α-bromoacetophenone (**I**) with the original morpholine-4-carbothionic acid hydrazide molecule (**II**). Progress of the cyclization reaction was monitored via thin layer chromatography (TLC) performed using a standard solvent system (butanol:acetic acid:water; 4:1:5) and Sifufol UV-254 TLC plates (Kavalier, Savaza, Czech Republic).

#### 4.1.1. Method for Synthesis of α-Bromacetophenone (**I**)

Acetophenone (0.3 mol) in glacial HOAc (200–250 mL) was stirred at 25 °C, and then treated dropwise with an equimolar amount of Br_2_. This mixture was first stirred for 1 h at 25 °C, and then again for 2 h at 50 °C–55 °C; after which, it was cooled, diluted with a 4- to 6-fold volume of ice water, and stored for 3–5 h. Following storage, the mixture was filtered to isolate a light gray residue, which was allowed to dry. Next, α-bromacetophenone was crystallized from 50 mL of absolute ethanol. The yield of the final product was 40%.

#### 4.1.2. Method for Synthesis Morpholine-4-Carbothionic Acid Hydrazide (**II**)

A solution of 55 g (1 mol) of potassium hydroxide in 50 mL of water and 1 mole of morpholine in 150 mL of ethanol was mixed with 1 mol of carbon disulfide with sufficient cooling to maintain its temperature at < 20 °C; this solution was then stored for 8 h. Next, dried potassium-morpholine-4-carbodithioate was added to a solution of 95 g (1 mol) of chloroacetic acid in 300 mL of water, neutralized with potassium carbonate. This mixture was stored for ~ 8 h. Then, a 50 mL volume of hydrazine hydrate was added, and the solution was evaporated to ~ 50% of its original volume. After cooling, morpholine-4-carbothionic acid hydrazide (II) crystals were formed, and the white solid precipitate was removed by filtration. The yield of morpholine-4-carbothionic acid hydrazide (II) was 79%. The isolated compound II was a white solid with the following characteristics: m.p.176–177 °C, ^1^H-NMR (DMSO-*d*_6_, 400 MHz, δ, ppm): 3.56–3.59 (m, 4H, 2CH_2_), 3.68–3.70 (m, 4H, 2CH_2_), 4.82 (s, 2H, NH_2_), 9.14 (s, br, 1H, NH). Anal. Calcd. for C_5_H_11_N_3_OS (161.23): C, 37.25; H, 6.88; N, 26.06. Found: C, 37.22; H, 6.85; N, 26.02.

#### 4.1.3. Method for Synthesis 2-Morpholino-5-phenyl-6*H*-1,3,4-thiadiazine Hydrobromide (compound **L-17**) (**III**)

α-Bromoacetophenone (20 mmols) in 20 mL of absolute ethanol was added to a solution consisting of 20 mmols of morpholine-4-carbothionic acid hydrazide (II) in 30 mL of absolute ethanol, and the mixture was refluxed for 20 minutes. After hot filtration and cooling, the white powdery precipitate was filtered and crystallized from absolute ethanol. The yield of 2-morpholino-5-phenyl-6H-1,3,4-thiadiazine hydrobromide was 75%. Compound III showed the following characteristics: m.p.191–192 °C, ^1^H-NMR (DMSO-*d*_6_, 400 MHz, δ, ppm): 3.81–3.97 (m, 8H, morpholino); 4.45 (s, 2H, CH_2_S); 7.5–8.0 (m, 5H, C_6_H_5_); Anal. Calcd. for C_13_H_16_BrN_3_OS (342.2): C, 45.60; H, 4.70; N, 12.30. Found: C, 45.70; H, 4.65; N, 12.25.

### 4.2. Biological Experiments

#### 4.2.1. Animal Preparation

Animal studies were conducted using healthy sexually mature, nonlinear albino male rats, mice, and guinea pigs obtained from the vivarium of the Institute of Immunology and Physiology of the Ural Division of RAS.

After arrival at the research facility, the animals were quarantined in the vivarium, and carefully observed to ensure they showed symptoms of any disease. All animals were housed under the same conditions, and fed according to a customary schedule. The protocols for all animal studies were approved by the Institute of Animal Care and Use Committee of the Institute of Immunology and Physiology of the Ural Division of RAS (F-1-2014-25). All studies were performed in accordance with guidelines developed at the European Convention for the Protection of Vertebrate Animals used for Experimental and Other Scientific Purposes (Strasbourg, France, 18.03.1986), APS's Guiding Principles in the Care and Use of Vertebrate Animals in Research and Training, and the Laboratory Practice Regulations of RF (Ministry of Public Health Order no. 267 from 19.06.2003) [30].

#### 4.2.2. Methods of Pharmacological Evaluation

Pharmacological evaluations were performed using methods described by E.F. Lavretskaya (1985), and in accordance with guidelines followed when studying the neuroleptic activity of drugs [14,31]. The injections of the compound **L-17** were made intraperitoneally. Injections were made in the lower right abdominal quadrant away from the midline to avoid inadvertent injection into the urinary bladder or cecum [32,33].

General locomotor activity, fear response, and affective aggression were evaluated in mice in an open field test chamber (Opto-Varimex, Columbus Instruments, Columbus, OH, USA) in accordance with standard protocols [14,31,34].

Changes in the apomorphine-induced stereotypy were evaluated in mice. The dopamine receptor agonist apomorphine was subcutaneously injected at a dose of 2 mg/kg, 20 min prior to testing [35,36,37].

Apomorphine-induced hypoactivity was evaluated on rats. The animals were placed in an open field chamber 10 minutes after administration of apomorphine (0.15 mg/kg) [14]. Low doses of apomorphine (0.01–0.15 mg/kg) were administered to induce chewing movements resulting from the effect on presynaptic dopamine receptors. Antipsychotic compounds inhibit the effects of apomorphine. This test is extremely sensitive and is used by investigators to identify new compounds that block presynaptic dopamine receptors [14].

The amphetamine-induced locomotion test was conducted with mice. Fifteen minutes after administration of amphetamine, the mice were placed into a cage containing a camera equipped with a multichannel video analyzer [14].

When administered to mice, amphetamine (phenaminum) causes an increase in spontaneous locomotor activity, which is associated with increased dopaminergic neurotransmission in the mesolimbic system of the brain. Typical neuroleptics inhibit the effects of amphetamine in a dose-dependent manner, whereas atypical neuroleptics have a weaker effect or fail to inhibit amphetamine-induced hyperactivity. In contrast, substances with antidepressant activity may enhance the effect of amphetamine [14].

Liver enzyme activity was evaluated by using the hexenal test to examine the hypnotic effect of barbiturates in mice. Many anti-psychotic compounds are known to potentiate the narcotic effect of barbiturates. In this assay, the test compound was injected intraperitoneally 10 min prior to hexenal administration (60 mg/kg, i.p.), and the percentage of dormant mice, and their time spent in a lateral position (in minutes) after administration of hexenal were recorded [38,39].

As there exists a subthreshold dose of hexenal that causes only a certain percentage of animals to sleep, increasing the percentage of animals that fall asleep after dosing testifies to an increased effect of the barbiturate. A test compound that fails to affect the duration of hexenal-induced sleep does not inhibit liver microsome activity, and can be classified as a "true potentiator" based on the classification system developed by Brodie BB (1955) [40].

Arecoline-evoked tremor was evaluated in mice to detect the central M-anti-cholinergic action of a compound. A single dose of the test compound was administered 30 min prior to a subcutaneous injection of arecoline (25 mg/kg); after which, the duration of tremor was recorded [14]. Anticholinergic activity manifests as a decrease in the duration or the complete elimination of tremor [14].

The effect of a test substance on the contractile activity of isolated smooth muscle organs (guinea pig ileum and rat seminal vesicles) was evaluated in rats by using the standard R. Blattner method with acetylcholine and epinephrine [17,18].

## 5. Conclusions

The relatively novel therapeutic concept of using combined autoreceptor antagonists that act on a1-adrenoceptors, serotonin, and choline receptors to treat cardiovascular diseases deserves further exploration in both preclinical and clinical studies.

## Figures and Tables

**Table 1:** Effects of 2-morpholino-5-phenyl-6*H*-1,3,4-thiadiazine (compound **L-17**) and Eglonyl on overall exploratory/locomotor activity, fear response, and affective aggression.

Test Sub.	(Dose mg/kg)/(N of Animals)	Explor/Locomotor Activ. for 10 m, M ± m	Fear Response and Affect Aggr., M ± m	Conventional Units, M ± m			
			Threshold, V	Squeak in Points	Decrease in Activity	Catalepsy	Analgesia
**L-17**	10/10				1.5 ± 0.07	0	0
	30/8				2.0 ± 0.05	0	2.0 ± 0.06
	60/10	104 ± 11 *	43 ± 1.5 *	0.5 ± 0.05 *	2.5 ± 0.05	0	2.5 ± 0.05
	120/10	15 ± 2 *	48 ± 1.5 *	0	3.0 ± 0.05	1.5 ± 0.05	3.0 ± 0.05
	240/10				4.0 ± 0.05	2.5 ± 0.05	3.0 ± 0.02
Saline 0.9%	/20	309 ± 26 *	40 ± 0.8 *	0.5 ± 0.07 *			
			30 ± 0.8	2 ± 0.05			
Eglonil	10/8				0	0	0
	20/8	250 ± 26	35 ± 0.5 *	0.9 ± 0.04 *	1.0 ± 0.05	0	0
	40/8	189 ± 12 *	38 ± 0.6 *	0.8 ± 0.05 *	2.0 ± 0.05	0	0.8 ± 0.05
	100/8				2.5 ± 0.06	1.5 ± 0.07	1.0 ± 0.05

* Differences *vs.* control were statistically significant at a *p*-value < 0.05. The severity of “squeak in points” (the equivalent of an "emotional" fear response) for stimulation with 20 V of electricity; “Threshold, V”—the threshold voltage at which the reaction approximates reactions of animals in the control group.

**Table 2:** Changes in apomorphine-induced stereotypy following administration of compound **L-17**.

Test Sub.	Dose, mg/kg	Apomorphine (2 mg/kg)			
		Latent Period Duration, sec., M ± m	Stereotyped Behavior Duration, min, M ± m	ED50, mg/kg	LD50/ED50
**L-17**	30	55.0 ± 9.8 *	22.5 ± 1.4	-	7.6
	60	67.5 ± 10.3 *	19.2 ± 1.0 *	80	6.9–8.4
	120	402.5 ± 50.0 *	5.8 ± 0.9 *	-	-
Saline		28.3 ± 3.6	23.2 ± 0.4	-	-
Eglonil	20	85 ± 4.7 *	15.6 ± 1.3 *	19	10.4
	40	150 ± 9.3 *	6.6 ± 0.9 *		8.9–12.1
Saline		42 ± 7.8	31.7 ± 1.8		

* Differences were statistically significant at a *p*-value < 0.05.

**Table 3:** Evaluation of apomorphine-induced hypoactivity following administration of compound **L-17**.

Test Sub.	Dose, mg/kg	Motor Activity (Number of Movemets for 5 min), M ± m
Saline	-	217.75 ± 17.10
Apomorphine		75.88 ± 19.33 *
**L-17** + Apomorphine (0.15 mg/kg)	40	26.4 ± 3.8 **
	60	16.50 ± 7.56 **
Eglonil + Apomorphine (0.15 mg/kg)	40	152.38 ± 12.42 **

* Differences were statistically significant at a *p*-value < 0.05., define; ** differences were statistically significant with apomorphine at dose 0.15 mg/kg.

**Table 4:** Results of the amphetamine-induced locomotion test after administration of compound **L-17**.

Test Sub.	Dose, mg/kg	Phenaminum (6 mg/kg)			
		Latent Period Duration, sec., M ± m	Stereotyped Behavior Duration, min, M ± m	ED50, mg/kg	LD50/ED50
**L-17**	50	247.5 ± 59.5	76.5 ± 5.5		
	100	308.3 ± 20.8 *	76.5 ± 5.5	170	3.5 (3.2–4.0)
	200	277.5 ± 25.9 *	60.3 ± 10.0		
Saline		185.0 ± 26.4	35.5 ± 5.3		
Eglonil	20	4.2 ± 2.2	70.7 ± 2.8	25	7.9 (6.7–9.2)
	40	5.2 ± 0.8	40.3 ± 1.8 *		
Saline		6.0 ± 1.65	118.0 ± 1.2		

* Differences were statistically significant at a *p*-value < 0.05.

**Table 5:** Results of the hexenal tests.

**Test Sub.**	**Dose, mg/kg**	**Hexenal (60 mg/kg)**			
		**Latent Period Duration, sec., M ± m**	**Sleep Duration, min, M ± m**	**ED50, mg/kg**	**LD50/ED50**
**L-17**	60	254.0 ± 75.0	65.7 ± 8.1 *		
	120	67.5 ± 4.2 *	96.0 ± 10.0	80	7.6 (6.9–8.4)
Saline		126.3 ± 6.4	39.3 ± 6.4		
Eglonil	20	168.0 ± 24.5	39.8 ± 1.7 *	42	4.7 (4.0–5.5)
	40	120.3 ± 15.8	40.0 ± 0.9 *		
Saline		150.2 ± 10.4	25.0 ± 0.8		
**Test Sub.**	**Dose, mg/kg**	**Hexenal (30 mg/kg)**			
		**Latent Period Duration, sec., M ± m**	**Sleep Duration, min, M ± m**	**The Number of Animals Fallen Asleep/Number of Animals in a Group**	
**L-17**	10	110.3 ± 70.8	12.8 ± 5.7	3/6	
	20	393.0 ± 51.4	28.0 ± 6.5	5/6	
	30	310.0 ± 95.0	24.2 ± 6.7	5/6	
	40	354.0 ± 30.2	50.2 ± 7.0	5/6	
	120	210.8 ± 57.5 *	68.5 ± 8.6	6/6	
Saline				1–6	

* Differences were statistically significant at a *p*-value < 0.05.

**Table 6:** The effect of compound **L-17** on arecoline-evoked tremor.

Test Sub.	Dose, mg/kg	Duration of Arecoline-Evoked Tremor, sec., M ± m
**L-17**	30	-
	60	14 ± 0.7 *
	120	14 ± 0.7 *
Saline		24 ± 1.5
Eglonil	20	15,5 ± 0.5
	40	17 ± 0.8
Saline		16 ± 0.9

* Differences were statistically significant at a *p*-value < 0.05.

**Table 7:** The influence of compound **L-17** on the contractile activity of isolated smooth muscles.

Object	Agonist	Concentr., mol/L	Eglonil		L-17 Compound	
			Amplitude, mm, M ± m	Diff. to Contr. Values, %	Amplitude, mm, M ± m	Diff. to Contr. Values, %
Rat seminal vesicles	Epinephrine 1 × 10^−5^ M	Control	57 ± 1	100	65 ± 3.4	100
		1 × 10^−6^	44 ± 1.2 *	77	58 ± 1.7	89
		Control	36 ± 2.3	100	60 ± 1.6	-
		1 × 10^−5^	22 ± 1.1 *	61	45 ± 2.4 *	75
		Control	24 ± 1	100	67 ± 3.1	-
		1 × 10^−4^	7 ± 1.3 *	29	23 ± 4.1 *	34
Ileum guinea pig	Acetylcholine 1 × 10^−5^ M	Control	46 ± 2.3	100	153 ± 6.3	100
		1 × 10^−6^	42 ± 1.5	91	150 ± 4.2	98
		Control	40 ± 1.7	100	150 ± 2.3	100
		1 × 10^−5^	38 ± 0.8	95	139 ± 3.1	93
		Control	37 ± 0.8	100	143 ± 2.4	100
		1 × 10^−4^	36 ± 0.9	97	130 ± 1.3	91
Ileum guinea pig	Serotonin 1 × 10^−6^ M	Control	105 ± 3.5	100	42 ± 4.5	100
		1 × 10^−6^	95 ± 4.0	90	38 ± 3.2	90
		Control	132 ± 2.4	100	46 ± 3.1	100
		1 × 10^−5^	73 ± 4.1 *	55	30 ± 2.4 *	65
		Control	61 ± 1.1	100	38 ± 2.1	100
		1 × 10^−4^	12 ± 0.8	20	20 ± 1.2 *	53

* Differences were statistically significant at a *p*-value < 0.05.
